# A Rare Case of Cholecystoduodenal Fistula Complicated With Gallstone Ileus and Upper Gastrointestinal Bleeding

**DOI:** 10.7759/cureus.24846

**Published:** 2022-05-09

**Authors:** Ching-Ching Chen, Sheng-Chun Wang, Tzu-Hung Chen, Shao-Jiun Chou, Hsiang-Chun Lan

**Affiliations:** 1 General Surgery, Cardinal Tien Hospital, Taipei, TWN

**Keywords:** gastrointestinal tract, fistula, gallstone, ileus, pneumobilia

## Abstract

Gallstone ileus is a rare presentation of cholelithiasis, which usually impacts the narrowest part of the bowel, the ileocecal valve. This occurs as a result of a bilioenteric fistula where a gallstone passed through and entered the gastrointestinal tract. It is most commonly encountered in elder patients and predominantly in females. Abdominal computed tomography is the investigation of choice for diagnosis in the majority of cases. Here, we present a 68-year-old female patient with a choledochoduodenal fistula complicated by upper gastrointestinal bleeding and gallstone ileus.

## Introduction

Gallstone ileus is a rare complication of gallstone disease. It occurs mostly after the formation of a biliary-enteric fistula, especially with the duodenum, which allows the passage of gallstones into the intestinal lumen, causing small bowel obstruction [1]. Bleeding from biliary-enteric fistula can also be found due to irritation by bile or gallstone passage. Surgical treatments may be in one stage or two stages. Many studies indicate that clinicians prefer deferred definitive treatment according to their own experiences [2].

Here, we present a rare case of choledochoduodenal fistula complicated by upper gastrointestinal tract bleeding and gallstone ileus.

## Case presentation

**Figure 1:**
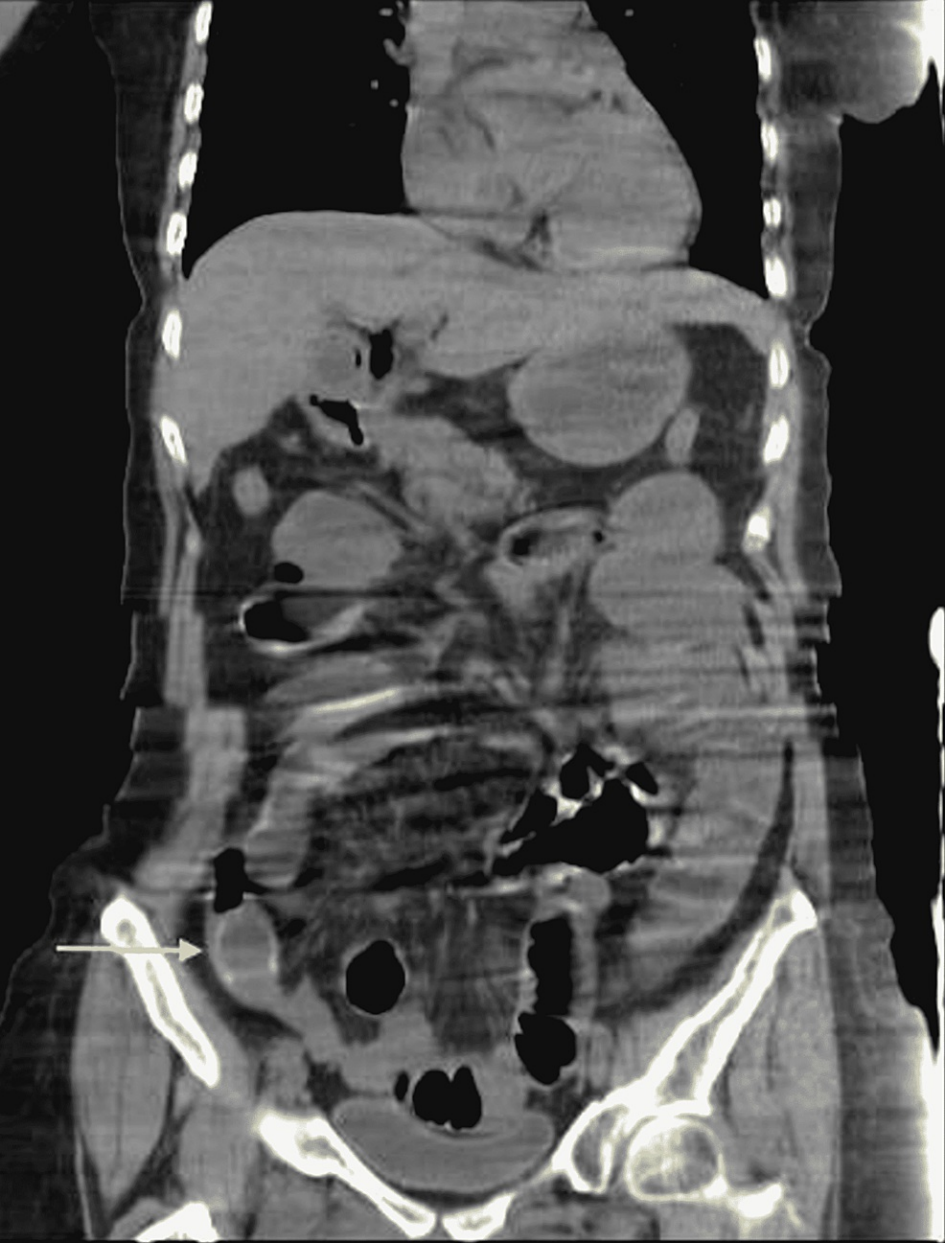
Coronal view of abdominal computed tomography Gallstone in the distal ileum.

**Figure 2:**
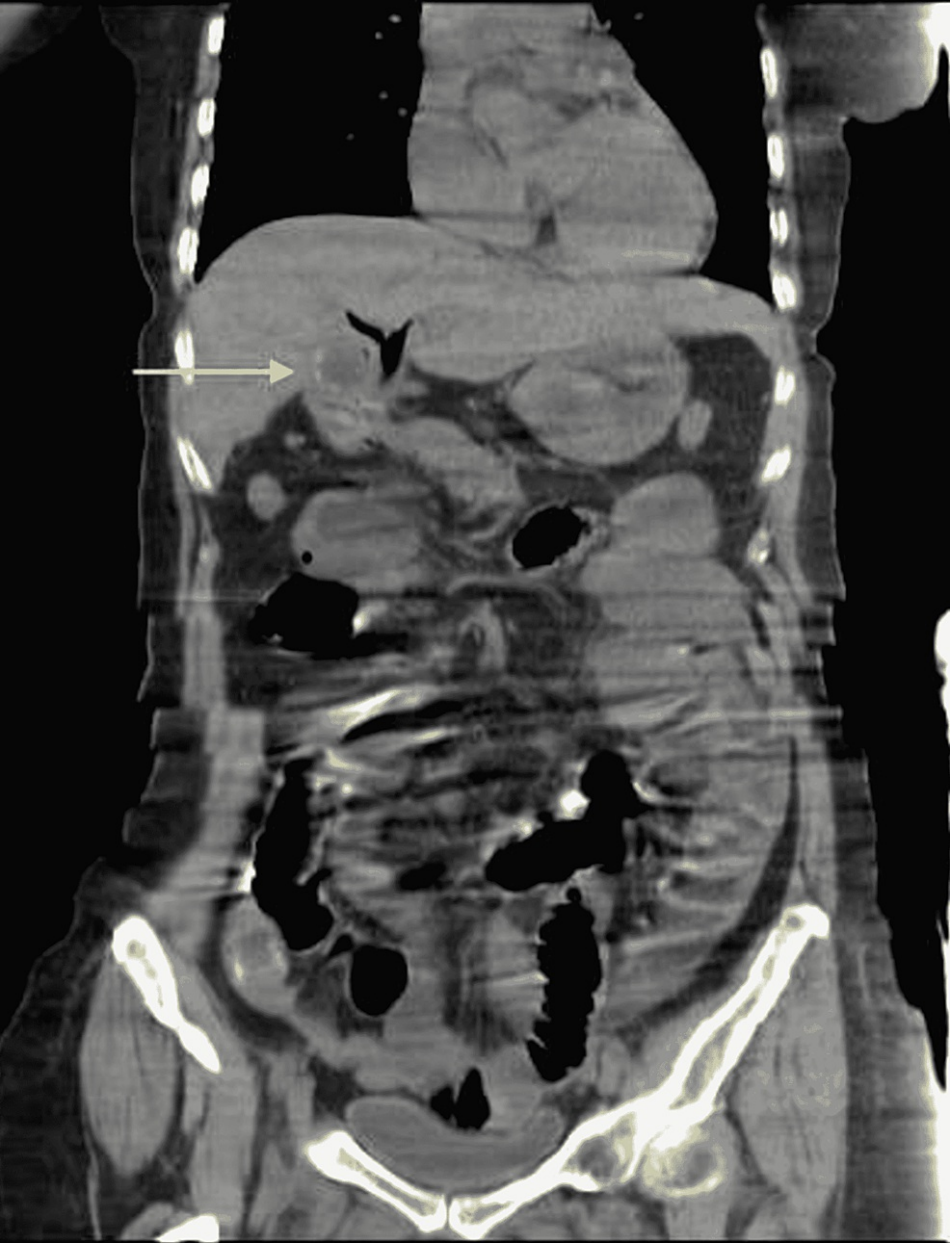
Coronal view of abdominal computed tomography Residual gallstone in the gall bladder with pneumobilia.

**Figure 3:**
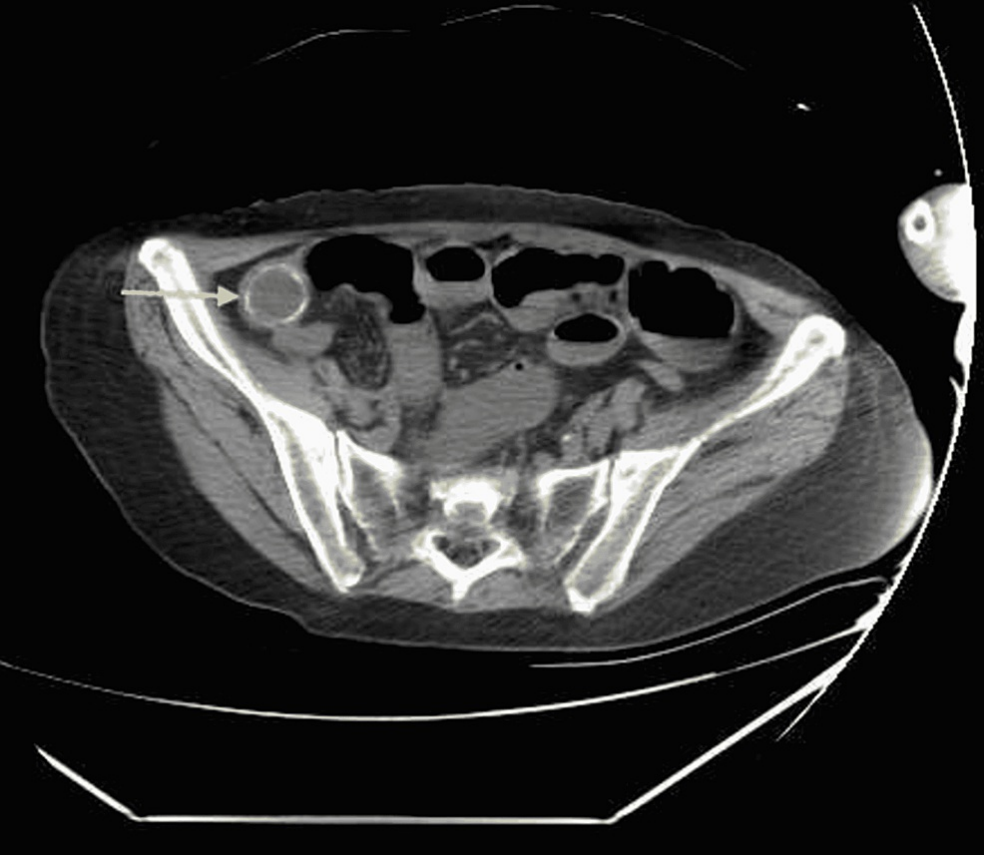
Axial view of abdominal computed tomography Gallstone in the distal ileum.

**Figure 4:**
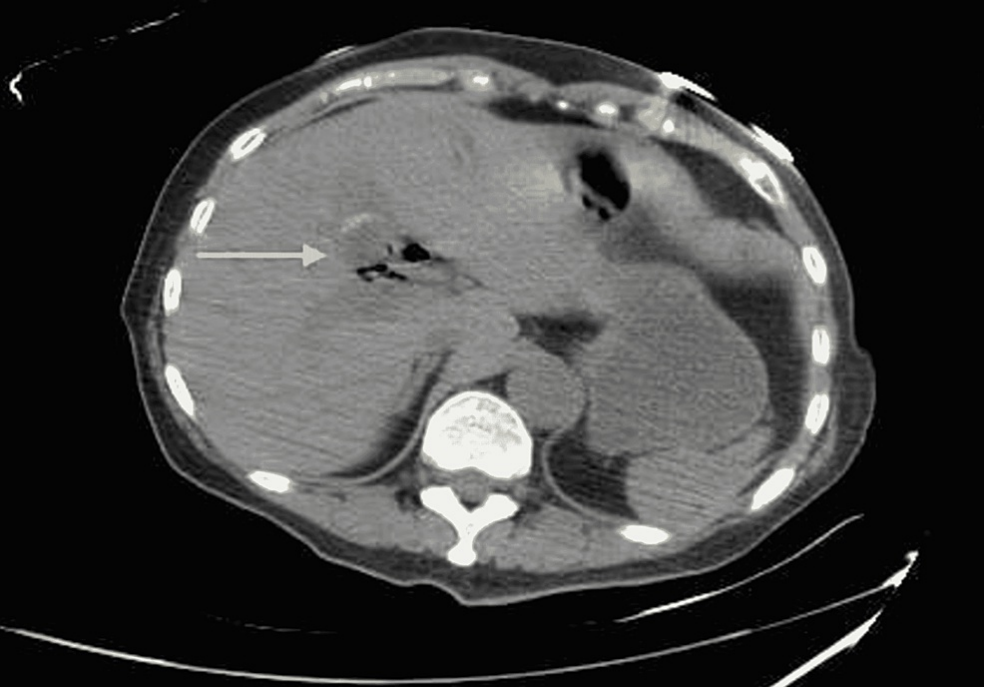
Axial view of abdominal computed tomography Residual gallstone in the gallbladder with pneumobilia.

The patient was a 68-year-old female patient without any prior history of systemic disease. The patient was brought to the emergency department due to changes in consciousness. The patient was ambulatory and able to self-care prior to this presentation. However, poor appetite and poor intake were noted as associated with vomiting and constipation for five days prior. There was no history of tarry stool or bloody stool passage before. Physical examination showed a distended abdomen with hypoactive bowel sounds. Other physical examinations cannot be performed due to hypovolemic shock with poor consciousness. Lab work revealed leukocytosis, severe anemia with a hemoglobin level of 4.9 gm/dl, and acute kidney injury. Computed tomography (CT) of the abdomen without contrast reported gallstone ileus and cholecystoduodenal fistula (Figures 1-4).

**Figure 5:**
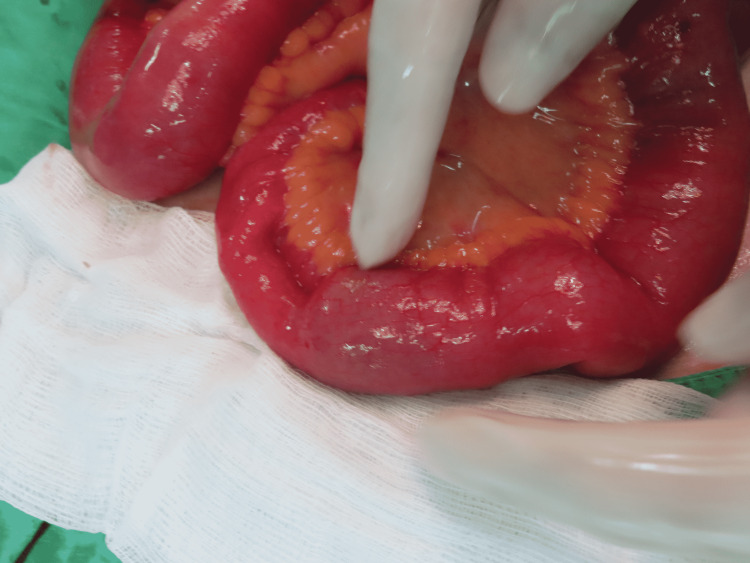
Operative finding Gallstone impaction in the distal ileum with proximal dilatation.

**Figure 6:**
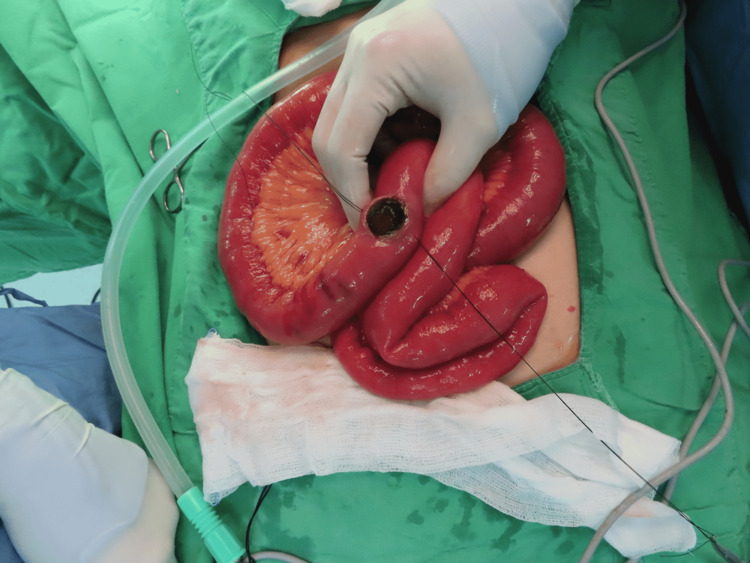
Operative finding Gallstone in the distal ileum (post-enterotomy).

After resuscitation including blood transfusion, an emergent exploratory laparotomy was performed. A large gallstone located in the distal ileum was found to have caused an intestinal obstruction (Figures 5, 6). Enterotomy and removal of the gallstone were performed.

**Figure 7:**
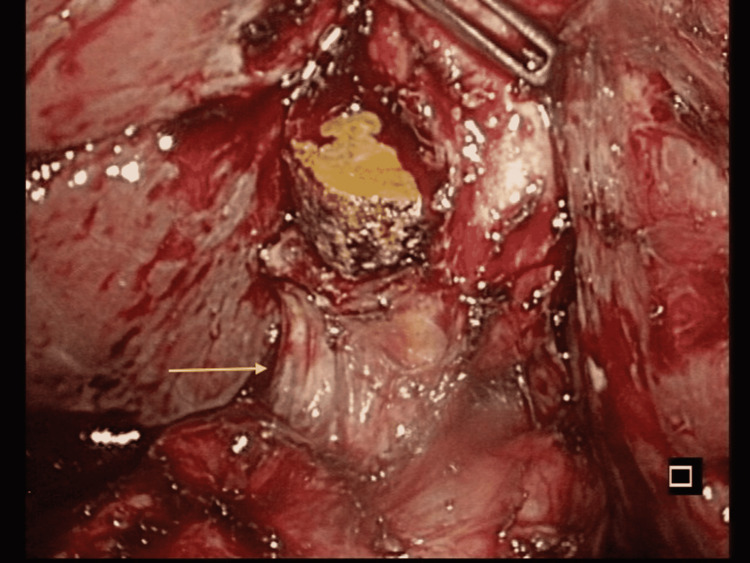
Laparoscopic finding Cholecystoduodenal fistula (arrow) with a residual gallstone in the gallbladder.

**Figure 8:**
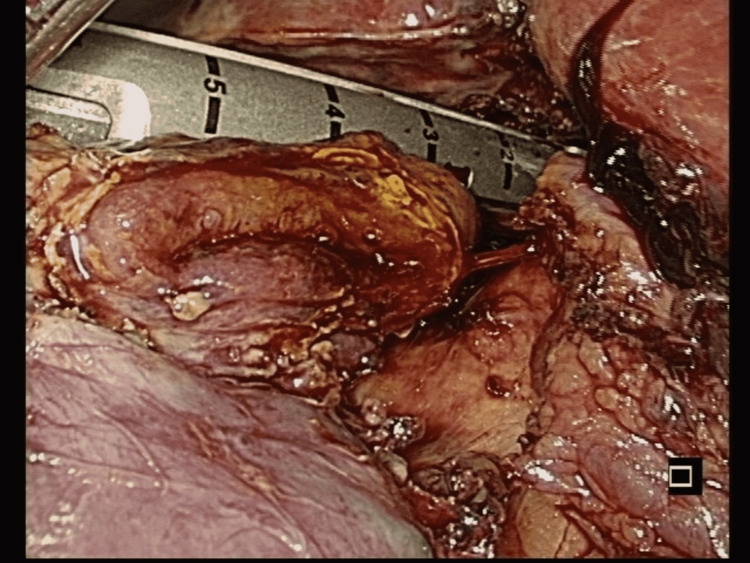
Laparoscopic finding Fistulectomy and resection of the duodenal stump with Endo GIA (Medtronic, Minneapolis, MN).

The patient's condition stabilized after the intestinal obstruction was improved. Upper gastrointestinal tract bleeding was confirmed by a bleeding scan, which reported increased tracer activity in the region of the upper abdomen with curvilinear movement through the small bowel. Interval operation of laparoscopic fistulectomy, cholecystectomy, and partial gastrectomy with Billroth II anastomosis for cholecystoduodenal fistula was performed one month after the first time operation (Figures 7, 8). Anemia due to upper gastrointestinal tract bleeding was also improved after the fistulectomy was performed.

## Discussion

Recurrent episodes of gallstone cholecystitis may result in larger gallstone formation and biliary-enteric fistula, complicated by gallstone ileus. The most common fistulas are cholecystoduodenal, and other types of biliary-enteric fistulas including cholecystocolic and cholecystogastric fistulas have also been reported. The most common site of obstruction is at the ileocecal valve, especially when the stone size is > 2.5 cm, suggesting a possible role of nasogastric decompression and fluid resuscitation in the management of gallstone ileus [3]. Prophylactic cholecystectomy is usually recommended in cases of large stones (>2.5 cm). However, this increases the unnecessary operative risks and costs for patients with asymptomatic gallstones. Although these patients are at risk of developing biliary-enteric fistula and gallstone ileus, the actual incidence of this progression is not known [4].

Gallstone ileus mostly presents in the form of small bowel obstruction, which is characterized as abdominal distension, colicky abdominal pain, nausea, vomiting, constipation, and features of dehydration such as feeling thirsty, dry eyes, dry mouth, deep color urine, and electrolyte imbalances. Although a history of previous biliary complaints is common in gallstone ileus, it is not a prerequisite. Only roughly 50% of gallstone ileus were noted with a history of previous biliary symptoms [5]. In some cases, gallstone impaction may cause pressure against the bowel wall and proximal bowel distension, resulting in necrosis of the bowel wall followed by perforation and peritonitis, which increase the mortality rate [6].

The typical radiographic features of gallstone ileus include small bowel obstruction, pneumobilia, and gallstone in the gastrointestinal tract that changes location on the serial film [7].

CT scanning is widely used as the investigation of choice in any other cause of acute abdomen, especially in intestinal obstruction with high sensitivity (90-93%) and specificity (100%) for gallstone ileus. In addition, CT scanning can also provide an accurate level of bowel obstruction that can be helpful in operative management and can further define the size and structure of the ectopic stone [8].

Upper gastrointestinal tract series using an oral contrast agent can also allow detecting the fistulous path extending between the gallbladder and the gastrointestinal tract, visualized as contrast accumulation within the gallbladder [9].

Cholecystoduodenal fistula rarely causes gastrointestinal bleeding, which occurs mostly due to invasion of the cystic artery by a duodenal ulcer or erosion by a gallstone. Bleeding associated with cholecystoduodenal fistula usually requires surgery because significant bleeding from the cystic artery is unlikely to be resolved by conservative management or endoscopic homeostasis [10].

The best management of gallstone ileus remains controversial. Enterotomy and stone extraction can resolve intestinal obstruction. Residual stones within the gall bladder may increase the risk of a recurrent attack of gallstone ileus, and even the risk of gallbladder cancer if recurrent cholecystitis has occurred.

For these reasons, two alternative methods of operation are introduced, which include a one-stage procedure as enterolithotomy, cholecystectomy, and fistula repair at the same time of operation and a two-stage procedure as enterolithotomy and interval cholecystectomy with fistula repair when the patient has recovered from the acute episode [11].

Reisner and Cohen compared mortality in patients who underwent enterolithotomy alone with those who underwent enterolithotomy, cholecystectomy, and fistula repair, which are one-stage surgery. The results showed that patients who underwent enterolithotomy alone had a mortality rate of 11.7% and patients who underwent one-stage surgery had a mortality rate of 16.9%. Although the recurrence rate for gallstone ileus is approximately 5%, enterolithotomy alone is the treatment of choice in gallstone ileus, especially in patients with hemodynamically unstable or significant comorbidities [12].

## Conclusions

Gallstone ileus and gastrointestinal bleeding are rare but important complications of cholecystolithiasis with cholecystoduodenal fistula. Good judgment in selecting the surgical procedure is required, especially in elderly patients with a high incidence of comorbidities. Two-stage procedures are the procedure of choice in unstable patients.
